# Characterization of NF-κB Reporter U937 Cells and Their Application for the Detection of Inflammatory Immune-Complexes

**DOI:** 10.1371/journal.pone.0156328

**Published:** 2016-05-27

**Authors:** Csilla Kecse-Nagy, Zoltán Szittner, Krisztián Papp, Zoltán Hegyi, Paolo Rovero, Paola Migliorini, Veronika Lóránd, László Homolya, József Prechl

**Affiliations:** 1 Department of Immunology, Eötvös Loránd University, H-1117, Pázmány Péter s. 1/C, Budapest, Hungary; 2 MTA-ELTE Immunology Research Group, H-1117, Pázmány Péter s. 1/C, Budapest, Hungary; 3 Institute of Enzymology, Research Centre for Natural Sciences, Hungarian Academy of Sciences, H-1117 Budapest, Magyar tudósok körútja 2, Budapest, Hungary; 4 Department of NeuroFarBa, Section of Pharmaceutical Sciences and Nutraceutics, Laboratory of Peptide and Protein Chemistry and Biology, University of Florence, Via Ugo Schiff 6, 50019, Sesto Fiorentino, Italy; 5 Clinical Immunology Unit, Department of Clinical and Experimental Medicine, University of Pisa, Pisa, Italy; 6 Department of Rheumatology and Immunology, Clinic Center, PTE, Pécs, Hungary; 7 Research and Development Laboratory, Diagnosticum Inc., H-1047, Budapest, Attila út 146, Hungary; Duke University Medical Center, UNITED STATES

## Abstract

Our study tested the hypothesis that immunoglobulins differ in their ability to activate the nuclear factor-κB pathway mediated cellular responses. These responses are modulated by several properties of the immune complex, including the ratio of antibody isotypes binding to antigen. Immunoassays allow the measurement of antigen specific antibodies belonging to distinct immunoglobulin classes and subclasses but not the net biological effect of the combination of these antibodies. We set out to develop a biosensor that is suitable for the detection and characterization of antigen specific serum antibodies. We genetically modified the monocytoid U937 cell line carrying Fc receptors with a plasmid encoding NF-κB promoter-driven GFP. This clone, U937-NF-κB, was characterized with respect to FcR expression and response to solid-phase immunoglobulins. Human IgG3, IgG4 and IgG1 induced GFP production in a time- and dose-dependent manner, in this order of efficacy, while IgG2 triggered no activation at the concentrations tested. IgA elicited no response alone but showed significant synergism with IgG3 and IgG4. We confirmed the importance of activation via FcγRI by direct stimulation with monoclonal antibody and by competition assays. We used citrullinated peptides and serum from rheumatoid arthritis patients to generate immune complexes and to study the activation of U937-NF-κB, observing again a synergistic effect between IgG and IgA. Our results show that immunoglobulins have distinct pro-inflammatory potential, and that U937-NF-κB is suitable for the estimation of biological effects of immune-complexes, offering insight into monocyte activation and pathogenesis of antibody mediated diseases.

## Introduction

Understanding immunoglobulin-mediated activation of myeloid cells is of great interest in the study of autoimmune conditions. Myeloid cells expressing receptors for the Fc part of immunoglobulins bridge adaptive and innate immunity. Different cell types in this group may mediate various effector functions through their Fc receptors (FcRs) after engagement with multimeric immunoglobulins (Ig). These include phagocytosis, expression of cytokines [1] and chemokines, as well as release of inflammatory mediators and reactive oxygen species [2]. In our work we focused on the nuclear factor-κB (NF-κB) transcription factor [3] mediated activation in monocytic cell line U937 [4] in response to immunoglobulin isotypes.

The system we applied here is based on the work of the Rosales group. They were the first to show, that among many other ligands, multimeric IgG (or mimicking it through cross-linking Fc receptors) initiates the signaling of the NF-κB pathway through FcRs in monocytes [5], and identified the steps leading to activation of genes under the control of NF-κB responsive elements [6].

The example of IgG-FcγR interaction illustrates well the complexity of various aspects that may affect the antibody triggered cell activation. Glycosylation [7], allotype [8],affinity and isotype distribution of antigen specific immunoglobulins along with Fc receptor polymorphism [9], Fc receptor copy number variations [10], and surrogate receptors [11] were all shown to be significantly modifying IgG-FcγR interaction, thus contributing to the fine-tuning of the immune response. This recent flare in our understanding regarding both immunoglobulin structure variety and its interaction with the immune system through Fc receptors, calls for the development of techniques capable of detecting immunoglobulins in a more biological sense.

Our hypothesis in this study was that measurement of activation levels induced by various immunoglobulins could help us (i) to reveal differences in their proinflammatory potential, and (ii) to detect the presence of antigen specific antibody response from autoimmune biological samples. For our investigations we used premonocytic cell line U937, a widely used model for monocytes. The receptor repertoire of these cells include receptors for IgG, such as the high affinity FcγRI and the low affinity FcγRIIA^R131^ [12], as well as FcαRI [13], the low affinity receptor for IgA. The IgG subclasses: IgG1, IgG2, IgG3 and IgG4, were shown to differ in binding to FcγRs. FcγRI can bind IgG1, IgG3 and IgG4 with high affinity, but not IgG2, while FcγRII^R131^ has lower affinity to IgG1, IgG3 and IgG4, and shows weak binding to IgG2 [14], yet their contribution to NF-κB activation remained unexplored. Microbial products are known to trigger NF-κB translocation in innate immune cells through recognition by pattern recognition receptors [15]. Bacterial lipopolysaccharide (LPS), a potent activator of NF-κB translocation in monocytes, as well as in U937 cells [16,17], signals through CD14 and toll like receptor 4 (TLR4) [18], which are both expressed on U937 cells [19] [20].

Rheumatoid Arthritis (RA) is a systemic autoimmune disease, where sustained inflammation can result in irreversible injury of joints and erosion of bone [21]. Among other characteristics presence of autoantibodies and elevated levels of various inflammatory cytokines are important factors of the disease. Anti-citrullinated peptide antibodies (ACPA) are found in the majority of RA patients and used as a biomarker of the disease [22], and their pathogenic role is also suggested [23]. Monocytes are suspected to play a central role in RA, by connecting autoantibodies to cytokine production, as well as by their potential to differentiate into osteoclasts, the effector cells of bone resorption. These cells are therefore suggested targets of RA treatment [24].

We stably transfected the U937 cell line with an NF-κB Responsive Element-driven GFP, and measured the production of green fluorescent protein (GFP) with different fluorescent methods upon activation by various stimuli with the focus on immunoglobulins to determine their inflammatory potential.

## Materials and Methods

### Serum samples and ethics statement

Serum samples from RA patients (n = 30) and healthy controls (n = 10) were obtained by venopuncture at University of Pécs and at University of Pisa (PISA) and were stored at -70°C until use. RA patients fulfilled the international criteria [25].

The relevant ethics committees of Hungary (Egészségügyi Tudományos Tanács, Tudományos és Kutatásetikai Bizottság) and Italy (Comitato Etico Area Vasta Nordovest, Azienda Ospedaliera Universitaria Pisana, Pisa) gave their approval for conducting study with the following contract numbers, respectively: 24973-1/2012/EKU (658/PI/2012.), 45066/2012. Written informed consent was obtained from all participants.

### Cell Culture

Human premonocytic cell line, U937 (ATCC® CRL-1593.2™) [4] and its stably transfected derivative, U937-NF-κB were cultured in RPMI-1640 medium supplemented with 10% fetal bovine serum (FBS), 2 mM glutamine, 100 IU/ml penicillin, 100 μg/ml streptomycin and 1mM Na-pyruvate. Cells were maintained in a humidified atmosphere (5% CO_2_) at 37°C. All materials were obtained from Sigma-Aldrich (St. Louis, MO, USA).

### Antibodies and reagents

Labeled antibodies: PE-conjugated anti-human FcγRI (clone 10.1, IgG1 isotype), anti-human FcγRII (clone FUN-2, IgG2b isotype), anti-humanFcγRIII (clone 3G8, IgG1 isotype), anti-human FcαRI (clone A59, IgG1 isotype), mouse IgG1 (clone MOPC-21) and mouse IgG2b (clone MPC-11) were purchased from Biolegend (San Diego, CA, USA).

Non-conjugated antibodies: anti-human FcγRI (clone 10.1, IgG1 isotype) anti-human FcγRII (clone AT10, IgG1 isotype) and anti-human FcγRIII (clone 3G8, IgG1 isotype), were obtained from Abcam (Cambridge, UK). Human IgG, human IgG1, human IgG2, human IgG3, human IgG4, human IgM, human IgA were purchased from Sigma-Aldrich, goat anti-human IgG fragment (GAH-IgG F(ab’)_2_) was obtained from Jackson Immunoresearch (West Grove, PA, USA).

LPS and bovine serum albumin (BSA) were purchased from Sigma-Aldrich. FcR blocking reagent obtained from Miltenyi Biotec (Bergisch Gladbach, Germany). Viral citrullinated peptide 2 (VCP2) was prepared by solid-phase peptide synthesis and characterized by MALDI-MS at the University of Florence. Hoechst 33342 stain was from Life Technologies/Thermo Fisher (Waltham, MA, USA).

### Stable transfection of U937 cells with a pNF-κB-GFP reporter plasmid

To generate U937-NF-κB, U937 cells were transfected as described by Ward et al. [26]. Briefly 10 μg pNFkB-PtGFP.2 (Xactagen, Shoreline, WA, USA) plasmid was added to 2*10^7^ cell in 400 μl RPMI-1640 without supplements in electroporation cuvette, the cells were electroporated at 960 μF and 0.17 kV. After selection with 1 mg/ml G418, cells were plated and resistant clones were isolated on the basis GFP production triggered by LPS.

### Characterization of receptor expression profiles in U937and U937-NF-κB cells by FACS analysis

1*10^5^ U937 and U937-NF-κB cells were stained with PE-conjugated anti-FcγRI, anti-FcγRII, anti-FcγRIII, anti-FcαRI, as well as IgG1 and IgG2b isotype controls for 20 min on ice, followed by one wash with phosphate-buffered saline (PBS) +1% FBS azide buffer and the cells were analyzed by flow cytometry. In all flow cytometry analysis 3*10^4^ cultured cells were measured by FACSCalibur (Becton-Dickinson, Franklikes, NJ, USA) and analyzed by FCS Express V3 (De Novo software, Los Angeles, CA, USA).

### *In vitro* activation of U937-NF-κB cells by immunoglobulin and LPS

Each cell activation assays were carried out in 96-well, flat bottom cell culture microplates (Greiner, 655180). In this experiment the surface was coated with 50 μl 10 μg/ml human IgG diluted in sterile PBS and incubated for 1 h at 37°C. Following this, the plate was washed twice with sterile PBS, and once with RPMI 1640 +10% FBS (medium). 1*10^5^ U937-NF-κB cells were added to the wells in 300 μl medium. For positive control 10 μg/ml soluble LPS, for negative control 10 μg/ml soluble human IgG was added directly to the cells seeded in uncoated wells. To block cell activation on human IgG-coated surfaces, prior to incubation with cells, the wells were treated with 10 μg/ml GAH-IgG F(ab’)_2_ in sterile PBS for 1 h, at room temperature (RT) to mask surface bound IgG, then washed twice with 100 μl PBS and once with 100 μl RPMI+10% FBS. The U937-NF-κB cells were seeded at a density of 1*10^5^ cell/well in 300 μl medium and were incubated for 24 h, at 37°C.

The activation was also measured on anti-FcγR-coated surfaces. 50 μl anti-human FcγRI, FcγRII or FcγRIII antibodies were immobilized in serial dilution from 20 μg/ml to 1.25 μg/ml in sterile PBS and incubated for 1 h at 37°C. After washing steps mentioned above, 1*10^5^ U937-NF-κB cells were added to the wells and analyzed by flow cytometry.

### Time- and dose-dependent activation of U937-NF-κB cells

The surface of 96-well, flat bottom cell culture microplates were coated with BSA, human IgG1, IgG2, IgG3, IgG4, IgA or IgM in two-fold serial dilutions from 20 μg/ml to 1.25 μg/ml in sterile PBS and incubated for 1 h, at 37°C. The plates were washed twice with sterile PBS, and once with RPMI 1640+10% FBS (medium). 1*10^5^ U937-NF-κB cells stained with 0.1 μM Hoechst 33342 were seeded in 300 μl medium. For positive control soluble LPS was added to the cells in serial dilution from 20 μg/ml to 1.25 μg/ml in medium. After a one hour long adhesion period, the cells were assessed by a high content screening system (HCS) in every 2 hours for an additional 12 hours (for further technical details see below).

### Blocking of U937 FcRs with human IgG subclasses

10 μg/ml human IgG4 diluted in sterile PBS was coated for 1 h at 37°C. The U937-NF-κB cells pellet were pre-treated directly with 10 μg/ml, 20 μg/ml, and 30 μg/ml soluble human IgG1, IgG3 or IgG4 for 20 min at 37°C, after incubation the cells were washed with medium The plate was washed twice with 100 μl PBS and once with 100 μl RPMI+10% FBS. The U937-NF-κB cells were seeded at a density of 1*10^5^ cell/well in 300 μl medium and incubated for 24 h, at 37°C and analyzed by flow cytometry.

### Cell activation by combination of human IgG subclasses and human IgA

Human IgG subclasses at 5 μg/ml concentration, diluted in sterile PBS were coated combined with 5 μg/ml or 10 μg/ml human IgA diluted in the same buffer. For controls the immunoglobulin subclasses were coated alone in a concentration of 5 μg/ml in sterile PBS. The U937-NF-κB cells were seeded at a density of 1*10^5^/well in 300 μl medium, incubated for 24 h, at 37°C and analyzed by flow cytometry.

### *In vitro* activation of U937-NF-κB cells by immune complexes

96-well, flat bottom plates were coated with 50–50 μl 20 μg/ml VCP2 peptide in sterile PBS and incubated for overnight at 4°C. After incubation, the wells were washed three times with 100 μl PBS, and blocked with 50 μl 3% BSA for 45 min at RT on orbital shaker. RA patients’ sera were diluted in 1:10 with 25 mM ethylenediaminetetraacetic acid (EDTA) contained 1% BSA and 50 μl diluted serum samples were added to each wells, and incubated for 3 h at RT on orbital shaker. Subsequently, the wells were washed twice with 100 μl sterile PBS and once with RPMI 1640+10% FBS. 1*10^5^ U937-NF-κB cells stained with 0.1 μM Hoechst 33342 were added to the wells, and incubated for 10 h at 37°C, then assessed by HCS equipment.

### HCS measurement and data analysis

The dose- and time-dependent assays, as well as immune complex-induced cell activation measurements were carried out with an ImageXpress® Micro XL (Molecular Devices, Sunnyvale, CA USA) high content screening system using a Nikon CFI Super Plan Fluor ELWD ADM 20x objective. The fluorescence signals for Hoechst (447/60 nm) and GFP (536/40 nm) were acquired at 377/50 and 482/35 nm excitations, respectively. The cells were kept at 37°C in a humidified atmosphere containing 5% CO_2_ throughout the measurements. Ten images per field with 2x binning were collected in both blue and green channels with 50 ms and 200 ms exposure times, respectively.

For analysis, MetaXpress® High Content Image Acquisition & Analysis Software Version 5.3 was used. The cells were counted in a size range of 6–35 μm. The geometric mean values of GFP signal in individual cells were analyzed by R version 3.2.0. The graphical evaluation of geometric mean values were carried out with GraphPad Prism 5.

### Microarray measurement and analysis

VCP2 diluted in MilliQ water to 4 mg/ml were printed onto 16-pad nitrocellulose-covered FAST slides (Main Manufacturing, Grand Blanc, MI, USA) by sciFlexarrayer S11 (Scienion AG, sciArraying service, Berlin, Germany) in triplicates, then stored at 4°C in sealed bags. Dried arrays were rinsed in PBS for 15 minutes before use, then 5-fold diluted serum, in 25mM EDTA, 0.05% Tween 20 and 5% BSA complemented PBS buffer was incubated at 37°C for 1 hour. Serum treated slides were washed with PBS containing 0.05% Tween 20 (PBS-T), then incubated with 1:2500 diluted DyLight 649-conjugated F(ab’)2 fragment goat anti-human IgG (Jackson ImmunoResearch) or with 1:1000 diluted Cy5-conjugated F(ab’)2 fragment goat anti-human IgA (Jackson ImmunoResearch). Labeling with fluorescent antibodies was carried out at room temperature for 30 minutes in PBS containing 5% BSA and 0.05% Tween 20. After washing in PBS-T, arrays were dried and scanned by FLAIR Fluorescent Array Imaging Reader (Sensovation). Data were analyzed by Array-Reader software by Sensovation, Radolfzell, Germany. Signal intensities were calculated by subtracting local background from medians of the three parallel signal intensities. Negative signals values were clamped to arbitrary value 1. Normalization procedure was carried out in two steps. First, slide to slide normalization was applied separately in the two printed batches. Slide specific normalization factors were calculated based on the signals in the subarray that was treated only with buffer but not serum sample. Human IgG and IgA features on arrays were used for normalization of IgG and IgA signals, respectively. This adjustment compensated for both overall biological variations in the samples and technical differences in the detection. A second normalization step was applied to compensate the possible differences in the two printing batches. Values in a given batch were divided by geometric means of values derived from the given antigen in control samples.

### Statistical Analysis

Mean ± SEM values were depicted on the graphs and unpaired Student's t test was used to evaluate significant differences as compare to the controls. For immune complex mediated cell activation analysis, groups were compared by the Kruskal-Wallis test followed by Dunn's multiple comparison test. The calculations were carried out with GraphPad version 5 using a significance level of 95% (*p<0.05).

## Results

### Cell surface receptor profile of U937 remained unchanged following transfection

**Fig 1:**
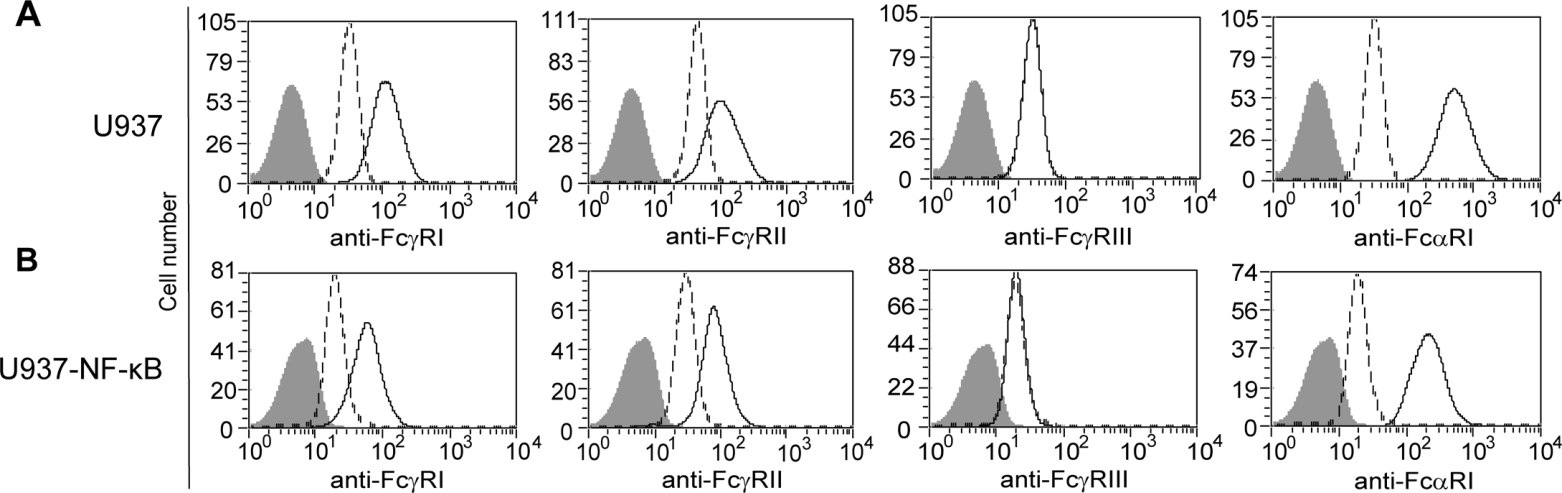
Expression of Ig receptors on the U937-NF-κB reporter cell line. U937 cells express FcγRI, FcγRII, FcαRI of Fc receptors (A). Transfection with NF-κB-GFP did not alter the Fc receptor expression patterns in the U937 cell line (B). U937 and U937-NF-κB cells were labeled with PE-conjugated anti-FcγRI, FcγRII, FcγRIII and FcαRI, antibodies. The histograms show the unlabeled cells (filled histogram), the corresponding isotype control labeled cells (dashed line) and the specific antibody stained cells (solid line).

Monocytoid cell line, U937 was stably transfected with a plasmid encoding NF-κB Responsive Element-driven GFP. After selection, cells were cloned and U937-NF-κB was generated. U937 cell expresses the high affinity FcγRI, the low affinity FcγRII for IgG receptors and FcαRI IgA receptor. U937 (Fig 1A) and U937-NF-κB (Fig 1B) cells were labeled with anti-FcγRI, anti-FcγRII, anti-FcγRIII and anti-FcαRI antibodies. The Fc receptor expression patterns remained unchanged in U937 cells after transfection (Fig 1).

### U937-NF-κB cells express GFP upon activation by LPS or IgG

**Fig 2:**
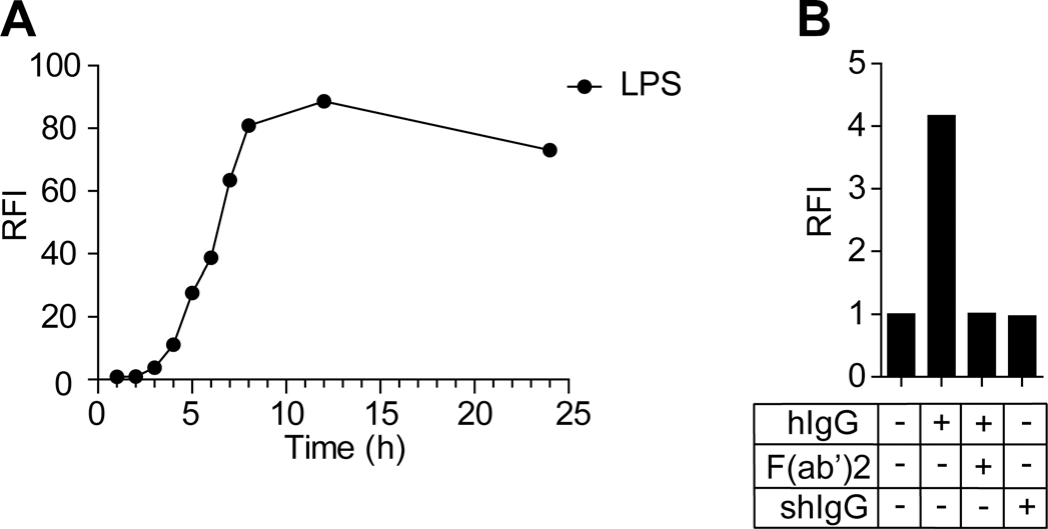
Detection of NF-κB activation by U937-NF-κB cells. Cells were treated with 10 μg/ml LPS (A), and the activation was detected for 24 h. Cells subjected to the medium only served as a negative control. The activation reached the maximum at 12 h. Cells were also activated on 10 μg/ml human immunoglobulin G (hIgG) coated surface (B). For negative controls, cells subjected to the medium only or to 10 μg/ml soluble human immunoglobulin (shIgG) on uncoated surface were used. The activation was tested on human immunoglobulin coat in the presence of 10 μg/ml goat-anti human immunoglobulin (F(ab’)_2_).

First, we tested U937-NF-κB responsivity with LPS, a widely used inducer of the NF-κB activation. U937-NF-κB cells expressed GFP in a time dependent fashion, reaching maximal fluorescence in about 12 hours, while untreated cells retain their baseline expression (Fig 2A). Next we investigated whether hIgG had the same activating effect on U937-NF-κB. Cells were incubated either on IgG coated surface or with soluble IgG. Immobilized IgG was indeed capable promoting GFP production in these cells, while IgG in solution triggered no activation. To verify that activation occurred through the Fc part of the antibodies, we tested cell activation following blocking the coated hIgG with anti-human IgG F(ab’)_2_ and found that this treatment completely abolished cell activation on the human IgG coated surface (Fig 2B). These data demonstrate that the NF-κB signaling pathway and the GFP expression were inducible by cross-linking the Fcγ receptors with immobilized immunoglobulin.

### Immobilized immunoglobulin triggers dose-dependent GFP production of U937-NF-κB cells

**Fig 3:**
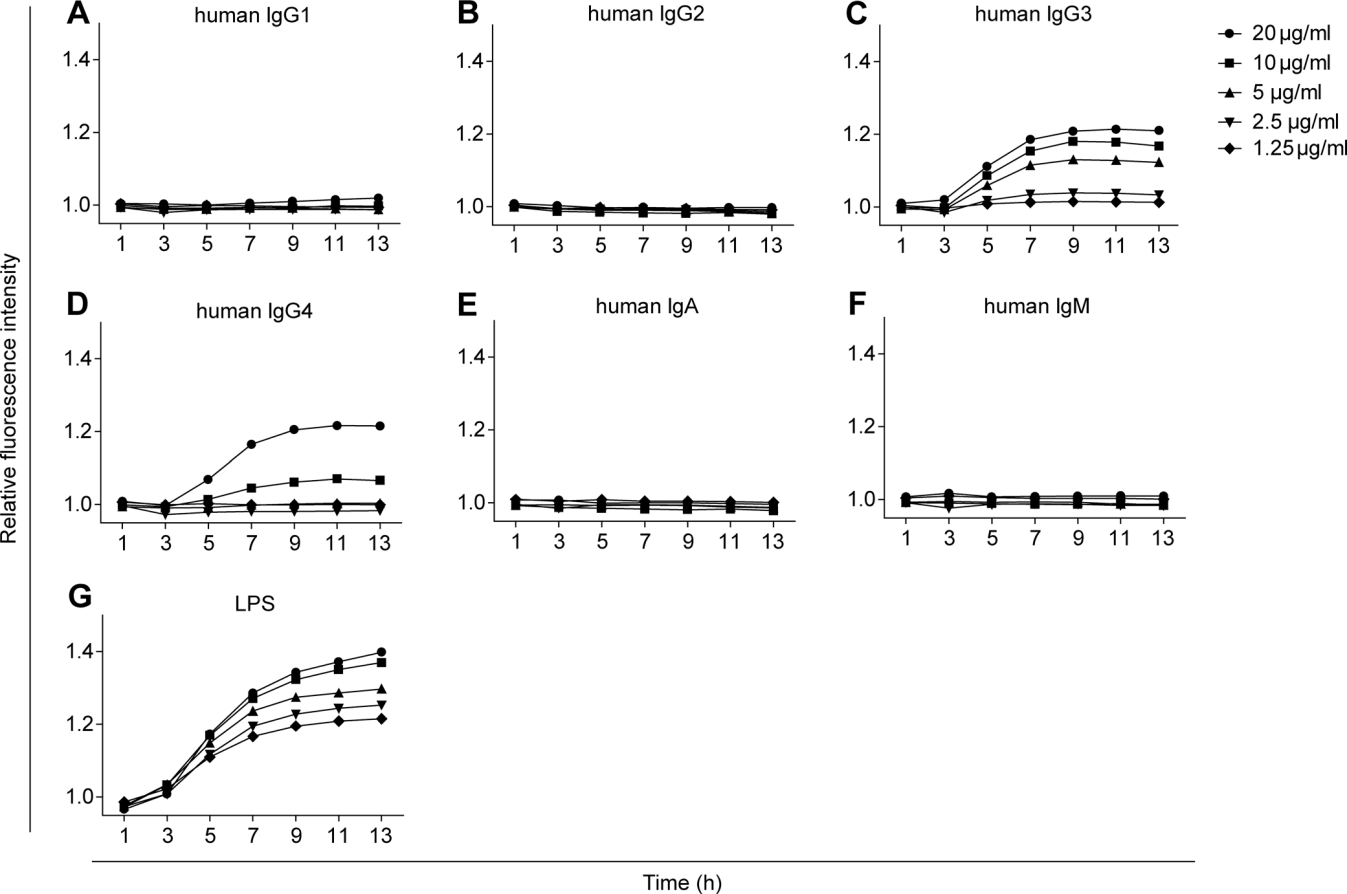
Time- and dose-dependent activation of U937-NF-κB cells. The activation of U937-NF-κB cells was screened on plates coated with BSA, human IgG1, IgG2, IgG3, IgG4, IgA, or IgM in two-fold serial dilution from 20 μg/ml to 1.25 μg/ml concentration. Following a one hour adhesion period, the cells were imaged for 12 hours by HCS (A-F). The results were normalized to the fluorescence intensities of cells measured on BSA coat. Soluble form of LPS served as positive control (G).

To see how human Ig isotypes activate GFP production, U937-NF-κB cells were incubated on increasing amounts of hIgG subclasses, hIgA and hIgM for 13 hours, their fluorescence intensities were recorded every two hours (Fig 3). Incubation of U937-NF-κB cells on surface coated with human IgG3 (Fig 3C) or IgG4 (Fig 3D) effectively activated GFP production in U937-NF-κB cells, while IgG1 (Fig 3A) had a very feeble effect. IgG2 (Fig 3B), IgA (Fig 3E) and IgM (Fig 3F) were ineffective. LPS-induced cell activation served as positive control (Fig 3G). The GFP expression on immunoglobulin coated surfaces reached its maximum after 10 hours.

### The activation of U937-NF-κB cells mainly occurred through the high affinity FcγRI

**Fig 4:**
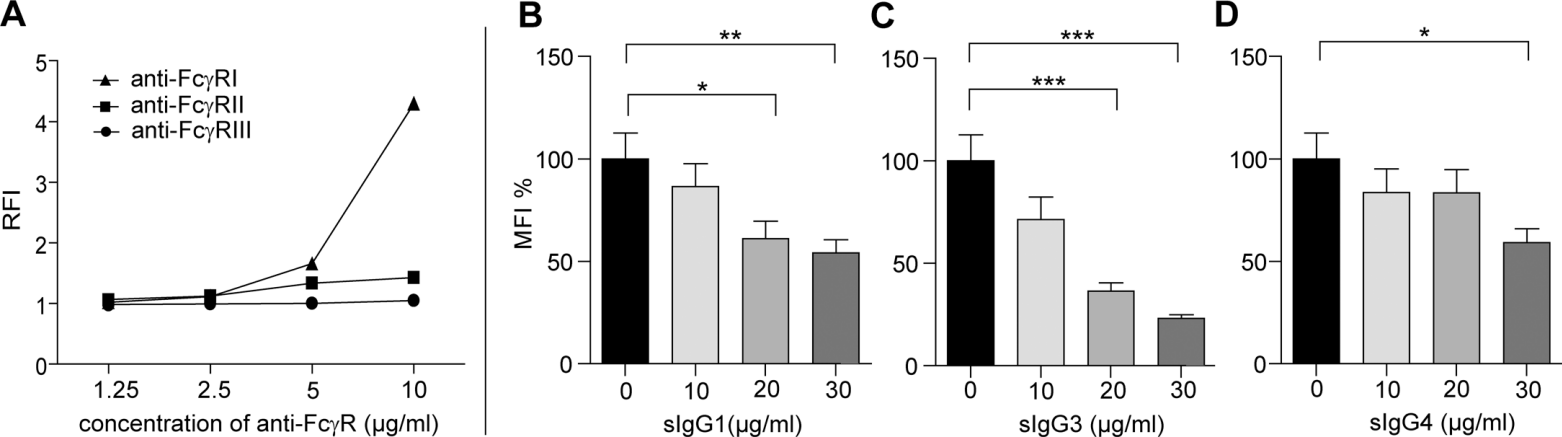
Activation of U937-NF-κB cells occurs mainly through FcγRI. The U937-NF-κB cells were activated on anti-FcγR-coated surfaces (A). FcγRI cross-linking induces strong, while FcγRII results in weaker activation. The cell activation was measured on IgG4-coated surfaces (B, C, D) in the presence of soluble IgG1(B), IgG3 (C) and IgG4 (D) in three doses (10, 20, 30 μg/ml). The untreated cells on IgG4 coat served as control (black bars).Results are mean % ± SEM % (n = 9) *p<0.05 versus control (untreated cells).

To distinguish how distinct FcγRs expressed on U937-NF-κB cells contribute to the activation of NF-κB pathway, we measured cell activation on immobilized monoclonal antibodies against FcγRI, FcγRII, or FcγRIII (Fig 4A). The activation by anti-FcγRI coat was proved to be the most effective stimulus as compared to anti-FcγRIII coat, which was used as a negative control. The anti-FcγRII antibody coated surface also enhanced GFP production, yet to a much lower extent than in the case of anti-FcγRI antibody, suggesting FcγRI to be more relevant in the FcγR-mediated NF-κB activation in the U937-NF-κB cells To further expand this observation we analyzed U937-NF-κB activation on immobilized IgG4 following incubation of the cells with soluble IgG1, IgG3 and IgG4 (Fig 4B). As FcγRI can bind monomeric IgG, we expected that its saturation would result in reduced GFP production on IgG4 coat because of competition between soluble and immobilized IgG. IgG4 has high affinity to FcγRI but not to FcγRII, thus, FcγRI is expected to trigger activation by IgG4. The affinity of FcγRI to IgG4 is slightly lower than to IgG1 and to IgG3 [14]. Soluble sIgG1 and sIgG3 reduced the cell activation on human IgG4 coated surface at smaller dose as compared to the effect of sIgG4. By increasing the dose of sIgG1 the activation was inhibited by 13% to 46%, in the case of sIgG3 the inhibition was 29% to 77%, whereas sIgG4 blocked the cell activation significantly only at the highest dose by 40%. These results suggest that the after partial saturation of the FcγRI, the receptor bound IgG molecules will compete with those in immune-complexes for FcγRI, thus only the IgG subclasses binding with higher affinity to FcγRI could trigger activation through these receptors.

### IgA shows synergism when combined with IgG

**Fig 5:**
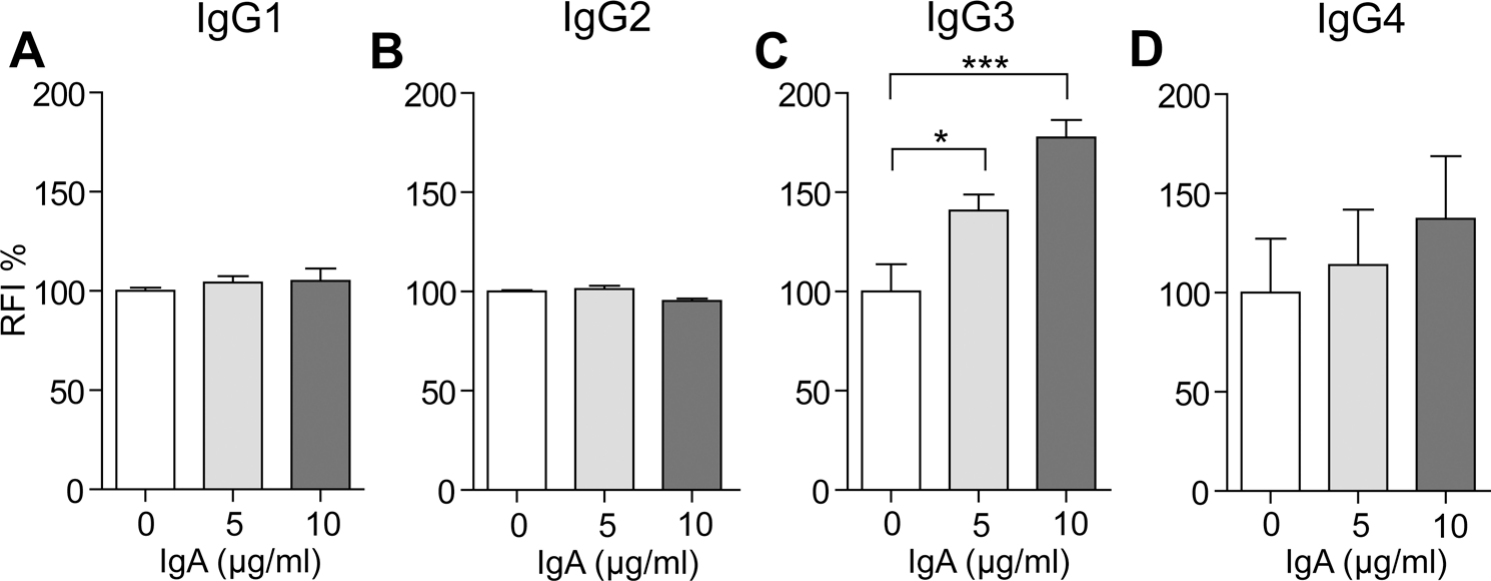
Activation of U937-NF-κB cells on IgG-coated surfaces in combination with IgA coat. U937-NF-κB cells were activated on surfaces coated with 5 μg/ml IgG1 (A), IgG2 (B), IgG3 (C), or IgG4 (D) and with 5 or 10 μg/ml IgA. The mean fluorescence intensities of the cells exposed to IgGs were normalized to the fluorescence intensities of untreated cells (medium only). For the analysis of combined IgG and IgA effects these normalized values were further divided by the respective IgA treatment values. Results are mean % ± SEM (n = 6) *p<0.05 versus control (without IgA).

As IgA alone did not activate the cells, we intended to determine, whether it had an effect in combination with activating IgG. We prepared combinations of IgG subclasses and IgA in various ratios. The activation significantly increased by combining IgA with IgG3, while with IgG4 the trend was obvious, yet non-significant (Fig 5). In the case of IgG1 and IgG2 coats, addition of IgA did not changed the activation of U937-NF-κB cells. Biological detection of autoantibodies using the reporter cell incorporates therefore the integrated measurement of IgA and IgG antibody isotypes that potentially trigger NF-κB activation and thus contribute to the inflammation in the body.

### NF-κB translocation mediated de novo protein synthesis triggered by by immune complexes is detectable by U937-NF-κB cells

**Fig 6:**
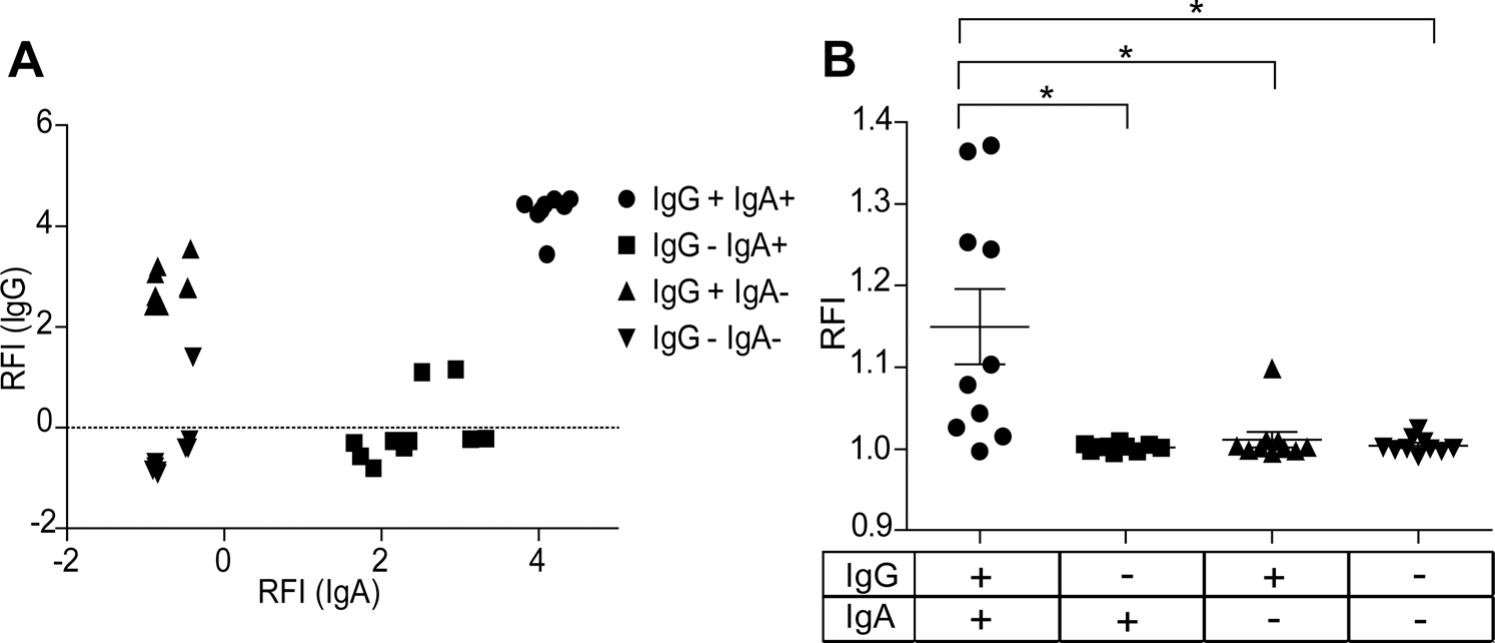
ACPA detection with the reporter cells. IgG and IgA reactivities of RA patients’ sera were measured for VCP2 peptide on protein microarray (A). The reactivity was analyzed with anti-IgG and anti-IgA antibodies, and the results were presented in relative fluorescence intensity (RFI). Ten times diluted RA serum samples were incubated on VCP2 peptide coat, and then U937-NF-κB cells were placed to the immuncomplex formed surface. The cell activation was normalized to the untreated cells (on BSA coat). Kruskal-Wallis and Dunn's multiple comparison tests were carried out to evaluate significant differences (*p<0.05).

To see how U937-NF-κB cells are capable of detecting activating immune complexes, we generated immune complexes by incubation of sera from rheumatoid arthritis patients with RA specific citrullinated peptide: VCP2. We selected the samples on the basis of their VCP2-specific IgG and IgA reactivity (Fig 6A), classifying them into four groups: double positive IgG+IgA+, single positive IgG-IgA+ or IgG+IgA-, and double negative IgG-IgA-. IgG+IgA+ samples were the most effective in activating U937-NF-κB cells, while one sample from the IgG+IgA-, and none from the IgG-IgA+ and IgG-IgA- were able to trigger GFP production in the U937-NF-κB cells. The generated reporter cells are therefore suitable for the detection of proinflammatory immune complexes.

## Discussion

The monocytic U937 cells, used here to model monocyte/macrophage responses, and the generated U937-NF-κB cells express two IgG receptors, FcγRI and FcγRII, and the IgA receptor FcαRI. It also known that they express the R131 variant of FcγRIIA [27], and a recent report shows that FcγRIIB is not expressed on this cell line [12]. Signaling via these receptors is expected to be responsible for our observations as have been shown previously for monocytes by cross-linking FcγRI and FcγRII [28]. To our understanding NF-κB activating properties of immunoglobulin isotypes have not yet been investigated. We first tested NF-κB translocation induced GFP production during incubation of U937-NF-κB on immobilized IgG. We verified that this activation is indeed mediated by the Fc fragment of IgG as (i) masking antibodies with anti-human IgG F(ab’)_2_ inhibited activation, and (ii) hIgG in soluble form did not induce activation. Immunoglobulin Fc part mediated cross-linking of FcγRI and/or FcγRII, thus, triggers signaling via NF-κB. As of note the monocyte inflammatory activation is not exclusively controlled by the NF-κB mediated production of cytokines, as FcyR mediated activation by IgG complexes was shown trigger caspase-1 activity, and thus may potentially contribute to the production IL-1 superfamily of proinflammatory cytokines [29].

The NF-κB transcription family is well-established as an important regulator of the inflammatory immune response [30]. The mechanism of its activation in response to TLR ligands has been widely studied [31]. Among other cell types monocytes and U937 cells were also investigated, and found to produce tumor necrosis factor-α (TNF-α), macrophage inflammatory protein 1-β, granulocyte colony stimulating factor (G-CSF) and interleukin-6 in response to LPS, supporting the inflammatory role of this pathway [12]. These well-known inflammatory cytokines are often found to be elevated in the sera of patients with autoimmune disease, such as RA [32]. Genome-scale location analysis of U937 after stimulation with LPS showed that more than 300 genes were affected in a response orchestrated by the five NF-κB family members [33]. While these studies focused on the binding of NF-κB transcription factor family members to their target sequences, here we also show the dynamics of the *de novo* protein synthesis during activation, as had been reported by others on luciferase based reporter assays with U937 [34,35]. In agreement with these results, U937-NF-κB cells, in response to LPS, produce GFP in a dose- and time-dependent manner.

In the current study we compared NF-κB translocation activating properties of immunoglobulin isotypes separately, which have not been investigated yet. Surprisingly, of the tested isotypes only IgG3 and IgG4 were found to have robust inflammatory potential, IgG1 had just a weak effect, whereas IgG2, IgA and IgM elicited no activation. It has to be noted that the variant R131 of FcγRII shows very little binding of IgG2, thus the relevance of our results regarding NF-κB activation by IgG2 is not yet conclusive. Investigations with cells expressing the FcγRII H131 variant is to be performed to verify this finding. However previously it has been shown that IgG2 immune-complexes can bind to FcγRI as well despite the low affinity of the FcγRI–IgG2 interaction [36]. Difference in glycosylation could offer an explanation, for the low activation by IgG1, yet the ability of IgG1 to compete with IgG4 (Fig 4) indirectly shows its binding to FcγRI, and thus, its weak activating property is not due to differences in glycosylation and FcγRI binding. In agreement with our findings, it has been shown *in vivo* that therapeutic IgG1 antibodies exert antibody-dependent cell mediated cytotoxicity (ADCC) and complement-dependent cytotoxicity (CDC) as effector functions, with a limited pro-inflammatory effect [37]. The highly activating, pro-inflammatory nature of IgG3 we found is in agreement with earlier findings showing this subclass to be the most effective in mediating effector functions. Until recently, IgG4 was thought to be highly inert, mostly because of disassembly of the constituting heavy chains, resulting in monovalent half-antibodies highly capable of neutralization just as in the case of its reported protective role in allergy. Recently, IgG4 triggered signaling has been shown [38], and the example of IgG4-related disease demonstrates that high local concentrations of IgG4 can be pathogenic and cause inflammation [39]. Our results also demonstrate the competition between the IgG subclasses for the high affinity Fc receptor (Fig 4). Presumably, this competitive saturation also happens *in vivo*, where during their trafficking to effector sites, FcγRI molecules of various cells will be saturated by IgG3 antibodies of various antigen-specificities, thus setting a threshold limit for local antigen specific IgG3 concentration to enable cell activation, offering a further explanation on the role of FcγRI in immunity [40,41].

Activation of the NF-κB pathway through FcγRs have been rigorously investigated in monocytes, and a signaling pathway independent of phagocytosis and actin cytoskeleton rearrangement was shown to signal through FcγRI and FcγRII [6]. These results suggest that affinity of immunoglobulins to FcRs does not necessarily reflect the inflammatory response mediated by these receptors. Lipid raft association of FcγRIIA was also found to be a perquisite of immunoreceptor tyrosine-based activation motif (ITAM) phosphorylation [42] and NF-κB activation through this receptor but not for phagocytosis [43], while FcγRI associates with the lipid-rafts without activation in basal state. Further investigations in U937 cells in response to activation by FcγRI and FcγRIIa lead to a conclusion that in monocytes, FcγRI shows enhanced contribution to the inflammatory response [12]. In agreement with these results we found that in the case of U937-NF-κB cross-linking FcγRs with anti-FcγRI to be a more potent activator of the NF-κB translocation induced GFP production compared to anti-FcγRII treatment.

U937 cells express no receptors for IgM, and in agreement with this, IgM did not activated the U937-NF-κB cells (Fig 3). FcαRI, the IgA Fc receptor was shown to be expressed in U937 cells, yet its NF-κB activating potential was not yet demonstrated to our understanding. Cross-linking these receptors with immobilized IgA did not induce GFP production. Priming of FcαRI through inside-out signaling by various cytokines, such as G-CSF and granulocyte macrophage colony-stimulating factor (GM-CSF) was shown to be necessary for ligand binding [44]. In agreement with this, IgA immobilized in combination with IgG3 and IgG4 enhanced the activation of the NF-κB pathway in a dose-dependent manner as compared to IgG3 and IgG4 coats only, while no effect was found for IgG1 and IgG2. Dai et al. have shown that stimulation of FcyRI in U937 cells results in the production of G-CSF, which was found to be an effective mediator of FcαRI priming in polymorphonuclear leukocytes [12]. Our results suggest that IgG by activating the NF-κB pathway through FcyRI can prime FcαRI, and thus, link antibodies of IgA isotype into the inflammatory response, as IgA was indeed capable of augmenting GFP production in U937-NF-κB cells. It has to be noted that the antibodies used in our study were monoclonals, and thus, does not necessarily mirror how the NF-κB activation happens, when stimulated with polyclonal isotype samples.

To further expand our observations and see, if U937-NF-κB cells can detect serum-derived antigen specific antibodies, we measured GFP production after incubation of VCP2 [45], a citrullinated peptide, with sera from RA patients. To ensure that complement does not play a role in the activation, we used EDTA-containing serum dilution buffer. As expected, neither IgG-IgA-, nor IgG-IgA+ group of samples triggered activation, verifying our previous finding that IgA alone cannot activate GFP production. Surprisingly, only one sample in the IgG+IgA- group responded; however, when both antigen specific IgG and IgA were present in the samples, we found a heterogeneous, yet significant elevation in GFP production (Fig 6). These results support that IgA can indeed act in combination with IgG during autoimmune reactions, and this synergistic effect of IgA and IgG can contribute to the inflammation through the NF-κB pathway. In agreement with our findings a pathogenic role for IgA in RA resulting in more severe symptoms has been suggested in the literature [46]. Moreover the possible role of ACPA pathogenicity in RA through activating monocytes and facilitating their differentiation into osteoclasts has been demonstrated [47]. Monocytes have been also showed as important effector cells producing inflammatory cytokines upon activation with ACPA in immune complex-based activation tests [48], establishing the importance of NF-κB translocation in RA pathogenesis.

Testing cell activating effector functions of ACPA-CCP (cyclic citrullinated peptide) immune complexes focused mainly on cytokine secretion of macrophages and monocytes so far (during incubation with the immune complexes). Laurent et al. showed that despite the significant alterations in FcγR level expression in monocytes from RA patients [49], monocytes from healthy controls and RA patients produce comparable levels of TNF-α upon induction by ACPA-CCP immune complexes [50]. Most of the studies to our understanding demonstrated this TNF-α production by monocytes in response to immune complexes formed with citrullinated fibrinogen [50–52]. Sokolove et al. proposed that this induction is the consequence of concomitant activation of the cells by the TLR4 ligand (citrullinated) fibrinogen and the FcγRs [52]. In agreement with these results a recent review proposes that cytokine production induced by ligand binding of FcγRs requires the presence of an additional danger signal [1]. However, when testing for TNF-α following stimulation of monocytes by immobilized IgG1 and IgG3, Magnusson et al. showed that monocytes produce TNF-α without CCP or additional activating signal [53].

Our results show how different antibody isotypes can control inflammation by activating NF-κB translocation through Fc receptors, demonstrating the role of IgG3 and IgG4, as well as the proinflammatory contribution of IgA in combination with these subclasses. We also verified previous data on the proinflammatory role of FcγRI, and finally demonstrated the diagnostic application of U937-NF-κB cells to detect VCP2 based autoimmune immune-complexes.
